# The Potential for Managed Alcohol Programmes in Scotland during the COVID-19 Pandemic: A Qualitative Exploration of Key Areas for Implementation Using the Consolidated Framework for Implementation Research

**DOI:** 10.3390/ijerph192215207

**Published:** 2022-11-17

**Authors:** Hannah Carver, Tessa Parkes, Wendy Masterton, Hazel Booth, Lee Ball, Helen Murdoch, Danilo Falzon, Bernie M. Pauly

**Affiliations:** 1 Salvation Army Centre for Addiction Services and Research, Faculty of Social Sciences, University of Stirling, Stirling FK9 4LA, UK; 2 Homelessness Services Unit, The Salvation Army, London SE1 6BN, UK; 3 Canadian Institute for Substance Use Research, University of Victoria, Victoria, BC V8P 5C2, Canada

**Keywords:** alcohol, alcohol dependence, homelessness, harm reduction, managed alcohol programmes, COVID-19, consolidated framework for implementation research, qualitative

## Abstract

People experiencing homelessness and alcohol dependence are at increased risk of a range of harms, including from COVID-19. Managed Alcohol Programmes (MAPs) are an alcohol harm reduction intervention specifically for this group. In this paper we report on qualitative findings of a mixed methods study investigating the potential utility of MAPs during the COVID-19 pandemic in Scotland. Interviews, conducted with 40 participants, explored potential views of implementing MAPs during the pandemic. Theoretically, we drew on the Consolidated Framework for Implementation Research (CFIR) to inform data collection and analysis. Six themes were identified which mapped onto three CFIR domains: perceptions of MAPs and the evidence base; necessary components of MAPs; changing culture of alcohol harm reduction; MAPs as a moral and ethical grey area; addressing a service gap; and securing buy-in and partnership working. Participants were generally positive about MAPs and viewed them as a key intervention to address a service gap. Several necessary components were identified for successful implementation of MAPs. Securing buy-in from a range of stakeholders and partnership working were deemed important. Finally, MAPs require careful, long-term planning before implementation. We conclude that MAPs are needed in Scotland and require long-term funding and appropriate resources to ensure they are successful.

## 1. Introduction

Alcohol dependence is defined as “craving, tolerance, a preoccupation with alcohol and continued drinking in spite of harmful consequences” [1]. People experiencing homelessness are at increased risk of developing alcohol dependence and associated physical (such as injuries, withdrawal seizures, liver disease, strokes and cancers) and mental health problems and social challenges, including housing, healthcare and financial [2,3,4]. Homelessness and alcohol dependence can often be connected, as alcohol can be the reason for someone becoming homeless or be a way of coping with current or previous challenges including social inequalities, stigma, and traumatic events [3,5,6]. While people experiencing homelessness are more likely to be dependent on alcohol, treatment options are limited, with abstinence-focused services being the norm [4]. Such services are not suitable for those who are unable or do not wish to stop drinking [7].

The situation for those experiencing alcohol dependence and homelessness was worsened by the COVID-19 pandemic in that, in the UK, the availability of alcohol, access to alcohol treatment and other services, and housing, all changed, particularly at the start of the pandemic in March 2020 [8,9]. Alcohol consumption increased among some of the general population and among many experiencing alcohol dependence [10,11]. Access to alcohol for those experiencing homelessness was impacted by factors such as shop closures, the use of credit/debit cards over cash, and loss of income from street begging, alongside related challenges regarding rules around wearing of masks, hand washing, social distancing, and self-isolation [9]. Alcohol treatment and associated physical and mental health services were impacted, with some services closing completely, others reducing dramatically, and many moving online [8]. Provision of housing also altered, with the rapid rehousing of people into hotels and increased shelter provision [9,12,13]. There were additional concerns for people experiencing homelessness due to the potential increased risk of contracting COVID-19 and becoming severely ill [14,15]. Thus, interventions and services which adequately address the needs of those experiencing homelessness and alcohol dependence were in much demand throughout the pandemic, and new possibilities opened for alcohol harm reduction given the pressure on treatment and other services.

Alcohol harm reduction is an appropriate alternative to detoxification and treatment, providing support and safer drinking options without the expectation or requirement of abstinence [7,9]. Managed alcohol programmes (MAPs) are an example of an alcohol-specific harm reduction intervention and have been specifically established for those experiencing homelessness and alcohol dependence. Eligibility criteria can very across programmes but commonly includes a history of chronic homelessness, regular public intoxication and illicit drinking, multiple attempts at abstinence-based treatment, and regular police and emergency department contact [6]. MAPs are an intervention providing alcohol regularly throughout the day, alongside a range of other supports including housing, healthcare, education and welfare [6]. Formal MAPs were first established in the 1990′s in Canada, with services being implemented more recently in the United States (US), Republic of Ireland, Portugal, and the United Kingdom (UK), with some of these opening as a direct result of the COVID-19 pandemic [9]. 

The growing evidence base for MAPs points to positive effects in terms of: reductions in police and emergency health service contact; reductions in non-beverage alcohol use and withdrawal seizures; less harmful patterns of alcohol consumption and lower overall alcohol consumption; and improvements in relationships and feelings of safety [4,16,17,18,19,20,21]. A recent scoping review identified the key components of successful MAPs, highlighting that these interventions can reduce alcohol-related harm and improve outcomes, although further research is required [22]. There is also some evidence to suggest that MAPs were beneficial during the COVID-19 pandemic in reducing harms associated with alcohol withdrawal, facilitating isolation/quarantine, and reducing the need for alcohol and COVID-19 related hospital admissions [23,24].

Previous research in Scotland has highlighted the need for MAPs to address the lack of alcohol harm reduction for those experiencing homelessness and alcohol dependence [25], and identified the key components for implementation of MAPs [26]. We build on this previous research to report on a study conducted during the early COVID-19 pandemic to explore the potential of MAPs as a response to COVID-19. At the time of this study no MAPS formally existed in Scotland, although some services operated informal MAPs both prior to and during the pandemic [9,25]. Since the study was conducted, the first MAP in Scotland was opened by Simon Community Scotland in late 2021 [27].

In this analysis we draw on the Consolidated Framework for Implementation Research (CFIR) [28] to provide insight into the key areas for implementation. The CFIR is a framework that includes the five domains of intervention characteristics, outer setting, inner setting, characteristics of individuals, and the process of implementation, with 39 constructs across the five domains [28]. Some domains and constructs may not be relevant to the specific intervention and in these cases do not need to be included [29]. While the CFIR has most commonly been used during or post-implementation [29], we used the CFIR pre-implementation of MAPs to facilitate identification of the key considerations and potential barriers prior to MAPs being implemented.

### Aims and Objectives

The aim of this mixed methods study was to explore the potential of MAPs in The Salvation Army (TSA) third sector (not-for-profit) homelessness services in Scotland during the COVID-19 pandemic, and specifically how MAPs might reduce infection risk for people experiencing homelessness and alcohol dependence. Initially the aim was to implement and evaluate MAPs in four TSA service settings, but it became apparent that doing so for a short period would be impractical and unethical. Therefore, the study was adapted to explore the views of clients, staff, and key stakeholders regarding how MAPs could be effectively implemented, and considerations that should be addressed prior to implementation. The study involved case record reviews, semi-structured interviews, meeting notes, and the creation of a series of paintings. The study methods have been reported in full previously [9]. Here, we describe the qualitative interview methods used as part of the analysis of potential implementation.

## 2. Materials and Methods

The study was conducted over a period of six months (May–November 2020, as per the funder’s requirement to conduct ‘rapid’ research) in four TSA services across Scotland that varied in size, geographical location, eligible clients, and staff. The study partnership between researchers and the TSA was supported by a wider research centre partnership that has been in place since 2017. This partnership allows research to be conducted that informs the work of the UK and Republic of Ireland based TSA. All four TSA services participated in interviews. Ethical approval was granted by University of Stirling’s General University Ethics Panel (GUEP; paper 917) and the Ethics Subgroup of the Research Coordinating Council of The Salvation Army (RCC-EAN200709). Risk assessments were in place for face-to-face data collection, as required by TSA and University of Stirling. 

Interviews were conducted with four groups: frontline staff; service managers; potential beneficiaries/clients (people accessing the services who would be eligible for a MAP); and external stakeholders (including those working in health, police, local/national government and other third sector organisations). Sampling was purposive to ensure a broad range of views/experiences in terms of gender, role, and organisation (stakeholders), gender and role (staff), and gender and levels of alcohol use (potential beneficiaries/clients). Participants were recruited in several ways. Service managers (those working in the four services and in other relevant roles in TSA) were involved in the study from the start and invited to participate in an interview. They were also asked to identify frontline staff and provided the research team with a list of potential participants (although the service managers did not know who was approached/interviewed by the research team). These staff were provided with information about the study by email. External stakeholders were identified via the research team’s networks as those with relevant expertise/experience and were invited to participate by email. Finally, potential beneficiaries were identified by staff in the four services and invited to participate in an interview. All participants were provided with an information sheet which detailed the interview process including confidentiality. 

All interviews were conducted between July and October 2020. Prior to the start of the interviews, written or verbal consent was provided by participants. Interviews with staff, service managers, and stakeholders were conducted online or by phone (by HC, PM, and WM), and potential beneficiary/client interviews were conducted by phone or in-person (by JD). The majority of interviews (39/40) were audio recorded and were an average of 51 min in duration. Different interview schedules were used for each group but focused broadly on perspectives on the implementation MAPs during the COVID-19 pandemic and were informed by the CFIR (see Supplementary File S1). At the end of the interview participants were thanked and provided with a debrief sheet which detailed further support and information. Potential beneficiary participants were provided with a £10 shopping voucher as a recognition of their time and expertise. Each researcher wrote detailed fieldnotes after each interview which enhanced reflexivity [30] and enabled changes to be made to the interview schedules. 

Audio recorded interviews were transcribed in full, with detailed notes taken for one unrecorded interview, and were all uploaded to NVivo12 (QSR International Pty Ltd., Doncaster, Australia, 2020) in four separate datasets, one for each participant group. Data were analysed using Framework analysis [31], a structured approach which is beneficial for policy-relevant research [32]. Transcripts were read in full and coded line-by-line by a team of four researchers (HB, JD, PM, TB), due to the number of interviews and the tight timeline of the study, with a fifth researcher checking coding for consistency. Data were coded both inductively and deductively (informed by the research questions), as well as being informed by the CFIR [28] which provided a way of identifying key factors influencing the implementation of MAPs. An initial thematic framework was developed (by HB and HC) after the coding of six transcripts which was used to code the remainder. Finally, the data were sorted into themes and sub-themes which corresponded with domains and constructs of the CFIR, and key quotes identified. 

## 3. Results

Forty interviews were conducted with external stakeholders (*n* = 19), service managers (*n* = 8), frontline staff (*n* = 7), and potential beneficiaries/clients (*n* = 6). Table 1 provides the characteristics of the participants.

The findings are presented as key themes which have been informed by three CFIR domains and various relevant constructs. Two of the domains (characteristics of individuals and process) were not relevant to the data collected. Table 2 provides details of the themes mapped onto three of the domains and relevant constructs. In this paper we report the novel findings although there is some overlap between these findings and those reported in our previous research on key components of MAPs [26]. 

### 3.1. Intervention Characteristics

In the CFIR, intervention characteristics refer to the main aspects of the intervention within the organisation which are required for successful implementation [28]. In this study this related to participant perceptions of MAPs and the necessary components that would need to be considered in the planning and delivery of MAPs in Scotland, both during the pandemic and longer term.

#### 3.1.1. Perceptions of MAPs and the Evidence Base

Generally, there were positive perceptions of MAPs, with a view that they were needed in Scotland, although some people had reservations, which will be discussed in more detail later in the paper. Many participants described the research from Canada as highlighting the strong evidence base, which demonstrated a range of potential benefits, including those relating to alcohol consumption/harm, health, criminal justice involvement, housing, and stigma. There was a view that evidence of the benefits of MAPs extended past the individual to public sector cost savings and wider societal benefits. Participants also noted that an additional benefit would be engaging with individuals who have had a long history of mistrust of mainstream services. While those working in the sector could see these benefits, there was a view that it was harder to convince the general public of the need for MAPs. Even with a strong evidence base, some participants expressed the view that the potential controversy of MAPs may be difficult to overcome:

*Within the field there is really compelling evidence, but I think in terms of the politics around it, there is a long way to go.*

(Stakeholder 2)

Many participants noted that, despite these positive views, the more limited evidence for, and acceptance of, alcohol harm reduction compared to that for illicit drugs influenced their perceptions of the potential value of MAPs. While participants noted a growing evidence base for MAPs from research conducted in Canada, there was a recognition that as no MAPs existed in Scotland at the time of the study, the lack of evidence in a Scottish context was described as a challenge:

*It will be new for us, you know, so sometimes it’s easier to sell something when it’s been running for ten years, and you’ve got a kind of whole package of data and information that you can say to somebody ‘oh we know how this works and we know that this happens’.*

(Manager 2)

Participants described a potential resistance to MAPs from several sources, including staff in services supportive of abstinence-based policies, local government, medical staff, and the general public, including those potentially living close to MAPs. This was for a range of reasons including the lack of a precedent for MAPs and a view that those experiencing alcohol dependence require abstinence-based treatments. Use of evidence was seen as an important way to overcome such resistance by demonstrating the need for MAPs and their effectiveness. Combining lived experience stories with rigorous research evidence concerning the potential beneficial outcomes of a MAP was viewed as important in this regard and perceived as a means of to securing funding and support. 

#### 3.1.2. Necessary Components of MAPs

Participants acknowledged several areas requiring decisions for successful implementation, including: choosing between different service models; tailoring to the local context; staffing; and funding. These necessary components will now be discussed in turn.

##### Different Service Models

Participants viewed MAPs as complex interventions with multiple considerations around choice of setting, models of care, approach to alcohol provision, eligibility criteria, and staffing (many of which have also been discussed in our previous research [26]). Participants discussed the possibility of both residential and drop-in models, and the benefits and challenges of introducing them in existing services, although residential settings were viewed as more suitable due to the provision of housing and round-the-clock support:

*If you are in a setting where maybe you’ve got access to just come down the stairs and talk to somebody at that point of really feeling like ‘I’m not doing so well here and actually I need a drink…or what I’ve had is not enough’. Whereas if it was a day centre it might close at five o’clock, so if somebody is feeling that at eight o’clock at night there just doesn’t seem to be that flexibility then for the individual who is on it… I think the setting that the MAP is in has to be flexible enough to kind of flow with the person… I think that would be quite difficult to manage in a day centre.*

(Manager 2)

According to participants, clear guidelines and rules would be required, and these would have to be consistent across different MAPs, as well as clearly explained to clients. Participants suggested that MAPs need to be well-structured, with rigorous clinical input/oversight, and a clear and consistent approach to clients who were not complying with programme rules, whilst remaining aligned with a harm reduction ethos. They stated that MAPs should be services that could address clients’ multiple and complex needs relating to their mental health, housing challenges and experiences of adverse events and trauma. Clinical oversight of clients’ alcohol use and physical and mental health was deemed important to ensure they were not deteriorating because of the MAP. 

The regular provision of alcohol within MAPs was viewed as a key component, providing individuals with a place of safety and stability. Within the MAP, clients would have a regular supply of alcohol and therefore the “*stress and anxiety*” of trying to find funds for alcohol each day would be reduced, providing “*a great sense of relief*” (Manager 4). There was a view that by providing regular doses of alcohol, clients would be better placed to consider their relationship with alcohol and their need for other support. Conversely, some participants expressed the view that clients should pay for their own alcohol, “*brings that level of stability… that would be beneficial*” (Manager 5), because it would help them to manage their own money and develop skills in budgeting. Others thought that alcohol would be provided as a, “*hook to get people engaged with the service*” (Stakeholder 17). Related to alcohol provision, practical considerations around alcohol licensing were highlighted as needing to be addressed as part of planning processes.

##### Tailoring to the Local Context

Participants noted that MAPs must be tailored to local context and need. They stated that, while MAPs have been successful in Canada and elsewhere, there are no guarantees of their effectiveness in Scotland. While several participants discussed issues relating to transferability, given the differences in client demographics, culture and context, they noted that MAPs could be adapted to the Scottish context by learning from the research conducted in Canada. Participants felt piloting a MAP with a targeted group could therefore demonstrate the potential benefits before implementing on a wider scale. There was also a view that MAPs would have to differ across different cities in Scotland:

*There is a huge cultural context to all of the work that we do, and I think if we were to do a MAP in [name removed, city A] and a MAP down here in [city B], they are not going to look the same because people in [city A] and people in [city B] are different. The demographics are really, really different. In [city A] there is probably around thirty or forty percent people from BAME [Black, Asian, and Minority Ethnic] communities, whereas in [city B] it wouldn’t be that way. So there needs to be a cultural context to the way that you do it.*

(Manager 1)

##### Staffing

Staffing of MAPs was also described as complex, with participants noting several concerns relating to training and resources. Participants stressed that MAPs would need to be adequately resourced. Suitable, well-trained staff would be required. One stakeholder reflected on the Canadian research on MAPs, noting that, “*the qualities and characteristics of the staff working there are very important*” (Stakeholder 3). Staff would also have to be provided with the necessary training and supervision to prevent burnout. Participants, particularly managers, reflected on the staffing challenges that were exacerbated by the COVID-19 pandemic, which meant that careful consideration would be needed to ensure delivery of a MAP would not add to an already overburdened staff’s workload:

*Most of the services run on a skeletal staff and that is because that’s how they are commissioned. You get a budget which is for staff and that is it… You cannae [can’t] just add something onto somebody else’s job. And effectively that is what we’d be doing with the MAP. So, I suppose even initially to have extra resource just to make staff feel supported in it. And it’s got a better chance of being delivered if people are in a positive way and no [not] feeling like it’s something we’ve added onto their to do list. I do think initially we would potentially need extra resource to appropriately manage it.*

(Manager 2)

There was also a view that there would have to be clear distinctions between the roles of service staff and clinicians, with third sector staff involved in the day-to-day running of the MAP and clinicians monitoring clients’ health:

*Staff would just be wondering what part they play. I think they would feel content supporting somebody as long as there was a clinical support that went along with it around… clear guidelines. We don’t live in a black and white world but people kind of like black and white guidelines. So, it’s like who buys the alcohol? What happens if a person goes out and drinks? For the staff, they would dae [do] anything really that would support an individual to make the changes that they want to make, as long as they have a clear understanding of a process.*

(Manager 2)

##### Funding

Discussions about the costs of delivering MAPs centred on securing funding, staffing, and purchasing of alcohol. There was no consensus regarding who would pay for clients’ alcohol, with participants noting the challenge of balancing clients’ needs and preferences with public perceptions of taxpayers’ money being used to purchase alcohol:

*There is research from Canada and other countries. There are different models so it’s not necessarily all government funding. There are cooperative models and there are various other ways of funding. People can pay for it themselves. But I think in the UK, people’s perception is that it would come under the NHS and as the NHS is free at the point of delivery, people then think it’s free alcohol and why should they get free alcohol? In terms of that hurdle of giving people free alcohol, as it is seen, that is still huge and is still a political battle that has to be fought and won.*

(Stakeholder 2)

Further challenges around securing funding were discussed in terms of the underfunding of alcohol services and in health and social care more generally, and what was described as ‘siloed’ funding which is split between health and housing (as MAPs would be). Participants explained that long-term funding would also be required for MAPs:

*Funding is obviously important, and you can find bits and pieces of money here and there but what people want is long term guaranteed funding and if we could make a case that health boards have to provide this then that reassures them and, you know, we do have to commit to funding these things.*

(Stakeholder 6)

The general view was that staffing would require significant investment, given the perceived complexity of delivery and the amount of support required by clients. Relatedly, costs associated with clinical input from healthcare professionals would also be required.

Given the complexity of MAPs and the wide range of necessary components identified, participants described a perceived need for the organization to adopt a well-planned, long-term approach to implementation. At the stage of study data collection, implementation in the TSA was only in the early discussion and conceptual stage. While rapid changes to the organization’s approach to alcohol use within services had been a response to immediate and unprecedented circumstances, i.e., from abstinence-only to a greater awareness and provision of harm reduction, participants felt that there was a need to take a slower, more deliberate approach to implementing a MAP longer term:

*Making sure that we are sufficiently resourced in it as well because sometimes we can just jump into things and sort of be building the ship as it’s being sailed so to speak. It’s really about having those resources in place.*

(Manager 3)

Initially, one or more MAPs were to be implemented during the early stages of the pandemic. However, it soon became clear that this would not be possible, nor would it be ethical to introduce them as a short-term intervention, given people’s needs for long-term housing and support. Therefore, participants noted that a slower approach to planning would allow MAPs to be suitable for people’s needs; to secure buy-in and funding; identify suitable sites and staff; and manage wider perceptions and expectations. While the COVID-19 pandemic was seen as accelerating the need for MAPs in Scotland [9], the general view was that planning and implementing such services would require a longer-term approach.

A number of necessary components were identified by participants which would need to be taken into account when designing and implementing MAPs, as well as building the evidence base in a Scottish context. Careful, long-term planning was described as essential, due to the complexity of MAPs and the wide-ranging decisions that would need to be made regarding implementation as there were no existing MAPs in Scotland at the time of this research.

### 3.2. Inner Setting

Inner setting describes the structural, political, and cultural context in which the intervention will be implemented [28]. The themes described below outline the current culture and climate in which the organization (TSA) operates, which in this case was of limited alcohol harm reduction approaches and MAPs as an ethical and moral grey area. 

#### 3.2.1. The Changing Culture of Alcohol Harm Reduction 

Participants discussed the need for a crucial culture change in the organization in order to implement MAPs: specifically, the move away from abstinence-only to being inclusive of harm reduction. Over the last decade, there have been shifts in organizational focus from abstinence-only approaches to alcohol and drugs to including harm reduction, which were accelerated by the pandemic. According to TSA participants, this enabled constructive dialogue in staff teams around MAPs and their potential implementation during the pandemic. The COVID-19 pandemic drew stark attention to a wider lack of harm reduction services for people experiencing alcohol dependence in Scotland, and the potential advantages of MAPs to address this, as one stakeholder noted:

*There was a worry that, you know, there is a risk here that lots of people could die if we don’t really start thinking and acting out the box and doing things a bit differently. And just because it’s such unusual circumstances that nobody has had to deal with before, it’s given a bit of freedom to try out things that maybe people would have been worried about before.*

(Stakeholder 5)

However, participants described service provision, within TSA and across the sector at the time of the interviews, as largely focusing on abstinence-based approaches or self-help. This was deemed unsuitable for some individuals, and often in contrast with the illicit drugs harm reduction ethos embraced across the sector. The current approach to alcohol use within TSA services was therefore stated by participants to require changes in practice and governance. Participants noted that, while alcohol use may occur in services, it was often ‘under the radar’, “*because there is a fear of making it public*” (Stakeholder 2). There was broad agreement about the need to account for the social and cognitive dimensions of alcohol dependence which would require longer term support, as well as a range of supports around physical and mental health and isolation. Participant discussion pointed to a perception that there was a greater need to attend to people’s reasons for drinking and the barriers and challenges they faced day-to-day:

*It shows you the great need and the gap in services. Where people are maybe getting detox, so the physical, you know, the physical effects of alcohol have maybe left them through like maybe Librium or Diazepam or what, but cognitively their relationship with alcohol is still the same.*

(Manager 4)

Throughout the interviews, those working in the organisation (TSA) described current examples of person-centred care which would extend to MAPs and were aligned with building a harm reduction ethos in services. They described compassionate staff, trusting relationships with clients, the need to give clients choice, responsibility and autonomy, and the need to involve clients in the development and running of MAPs. Participants discussed that staff and clients should work together to determine how a MAP would operate on an individual level. Potential areas of collaboration included dosage and type of alcohol consumed, goals (whether to reduce, aim for abstinence, or not reduce at all), and supports needed in other areas. 

Participants also stressed the need to trial interventions like MAPs given the lack of suitable, non-abstinence-based support for those experiencing alcohol dependence. Additionally, the novelty of MAPs was viewed as a potential challenge to implementation, with participants noting the need to demonstrate likely benefits over other more established approaches:

*It’s a relatively new idea. If you don’t work in this field, it is kind of counter-intuitive, the way that we are going to help people with addiction problems is by providing them with their substance of choice.*

(Stakeholder 13)

Thus, MAPs would sit within a culture that is beginning to see the value of alcohol harm reduction and the need for innovative approaches, but there was a view that more work would be needed to change current practice to enable MAPs to operate. 

#### 3.2.2. MAPs as a Morally and Ethically Grey Area

For some frontline staff, as well as those in very senior management in the organisation, the idea of MAPs was controversial, going against their ethical orientation and views of abstinence-based approaches to alcohol treatment. On the other hand, some viewed it as morally important and potentially lifesaving. Participants stressed the importance of being able to meet people where they were at and saw MAPs as a person-centered approach which could build on clients’ strengths, as discussed above. Staff teams had a varied response to the organisation’s change in approach to alcohol use within services during the pandemic, with one participant noting that it had caused division amongst some teams. These varied opinions on alcohol harm reduction were due to concerns about personal responsibility and roles; differing understandings of what a MAP is and how it would work; and a having a background in abstinence-based approaches:

*As a member of staff, I would be apprehensive if it was just like I don’t know right we are doing this, but you would want to know how it works, do you know what I mean? And how that would be monitored and how you would keep a service user safe and yourself?*

(Staff 4)

*There is (sic) so many questions. We were thinking… if we did that… in a residential or in a day centre, you know, like it’s a, we don’t feel as staff able to decide… how much alcohol this person needs, you know, how often, you know, or how do you spread it through the day or, given their need, you know? Like we wouldn’t have a clue about that so it would need to be linked closely with medical staff.*

(Staff 5)

The challenges of embracing harm reduction were particularly felt by staff with lived experience who had joined the organisation because of its abstinence-based approach:

*Staff have traditionally worked in ways where alcohol wasn’t allowed into buildings on the whole and well you suggest the fact that alcohol needs to be consumed in order to maintain safety within a building. That’s really like the word safety and alcohol don’t really go together for the organisation. They see that alcohol means unsafe, means dangerous, means chaotic. And I think one of the groups of staff who have kind of struggled with that change in practice possibly the most are those who may have had lived experience and have come to the organisation because of its stance on abstinence.*

(Manager 1)

Some participants discussed the challenges for the organisation as a whole, and the moral/ethical grey areas in which MAPs were situated. For example, participants noted potential issues relating to: the damaging effects of alcohol; the provision of alcohol to people with pre-existing health conditions such as liver damage; the potential blame placed on staff when things go wrong; and the general view that it is unethical to give, “*alcohol to… an alcoholic*” (Manager 1). The following quote describes some of these concerns:

*Fear. Particularly around ethical issues. For example, what about existing illness? How can that be justified giving more alcohol when someone has a pre-existing medical condition? Are staff vulnerable in regard to making those choices e.g., if they give alcohol and the person comes to harm? Ethically how does that work? If a person has a seizure and the staff member is responsible, how are they then protected? If someone has existing comorbidities, who is going to work out what to do to as they shouldn’t be given alcohol. Will alcohol cause related brain damage, will that be more harm than withdrawal? These questions are raised a lot.*

(Stakeholder 14)

A number of participants discussed concerns that MAPs may be used to keep clients, “*out of sight, out of mind*” (Stakeholder 9), and to allow them to continue drinking without trying to encourage them to reduce or eventually stop using, with some describing MAPs as palliative care. This was problematic for some participants, as it was felt that services should always have the longer-term aim of supporting people to stop drinking at harmful levels (which would not necessarily be the aim/focus of a MAP):

*Any programme for an addiction, it’s about reducing the harm and reducing the level preferably to none, I always think that should be the goal and I appreciate that not everybody is going to get there… I don’t think we should be about maintaining somebody on a harmful substance, it should always be working to reduce to nothing.*

(Stakeholder 1)

*We will just keep on giving them alcohol to maintain that dependent state and they will die prematurely, it’s almost like palliative care.*

(Stakeholder 9)

Despite some having reservations about the ethics of MAPs, others saw the relational side of MAPs as particularly important and in keeping with TSA’s person-centred values:

*It’s about us walking alongside the person. It’s not about us telling them how to go here, there and x, y, and z. There is no ulterior motive, we just want to see the person getting on, do you know what I mean? It’s like that unconditional positive regard, do you know what I mean? That’s the approach that we would be wanting our staff to take. It doesn’t matter how many times you’ve fallen we will still be here for you.*

(Manager 4)

The COVID-19 pandemic highlighted challenges for those experiencing homelessness and alcohol dependence and accelerated the work already being done regarding harm reduction involving changing policies and approaches within the organisation in order to keep people alive. For some, these changes were described as a, “*great relief*” (Manager 4) as staff were able to stop dangerous withdrawal symptoms by providing alcohol. Participants expressed the view that MAPs required a mindset shift, given that the organisation and many alcohol services in Scotland are traditionally abstinence-based. Thus, the move towards harm reduction and the impact on people’s values and beliefs likely occurred at a faster rate as a result of the pandemic. In summary, the data highlight a tension between abstinence-only and harm reduction approaches. Participants noted that more work would be required to change the mindsets of those working in the organisation, the wider field, as well as of the general public and politicians, to embrace harm reduction and enable MAPs to be implemented effectively.

### 3.3. Outer Setting

Outer setting describes the economic, social and political context in which the organisation delivering the intervention sits [28]. In this study, this related to the wider context and two themes were identified: addressing a service gap and securing buy-in and partnership working. It is important to note that there is some overlap here between inner setting and outer setting due to the size and complex structure of the TSA.

#### 3.3.1. Addressing a Service Gap

A number of participants noted that, while alcohol harm reduction was still controversial in Scotland, there was a growing understanding of a harm reduction ethos as a crucial rationale for MAPs and a framework for implementation. MAPs were described as providing non-judgmental and non-conditional support and thereby filling a large gap in service provision for those unable to abstain from drinking. This gap was further illuminated during the COVID-19 pandemic when there was a tension between people requiring access to alcohol to prevent withdrawal and being unable to leave their accommodation during periods of lockdown:

*We’ve been speaking to hostels and hotels and everyone else and saying ‘actually we can’t advise that they stop their drinking because it’s dangerous, you need to just continue drinking, you know, at whatever level is safest for them’ because sometimes that is the only thing that is practically possible. So that has been quite difficult for us to get that message out. We’ve seen lots of new innovations too… they are doing detoxes in slightly different ways with modified protocols. And then we’ve seen the really good work that has been done to get rough sleepers in and looked after that helps their physical health, it helps their nutrition… But we haven’t quite ticked the box around, you know, addressing their alcohol, and that to me is the next thing.*

(Stakeholder 7)

Clients not having to go out and look for funds for alcohol was described as a potential benefit, allowing them time to work with staff on their wider physical and mental health, social, and financial needs. 

Participants discussed at length the impact that MAPs could have on those who experience both alcohol dependence and homelessness who have been negatively impacted by the COVID-19 pandemic and the lack of suitable services to meet their needs. Client participants talked about their experiences with alcohol, including: difficulties during the early pandemic; the serious negative consequences attributed to their alcohol use; previous experiences of treatment; and how MAPs might address these challenges. They described drinking high quantities of alcohol every day, as reflected in the quantitative findings in our related paper [9]. Clients would usually fund their alcohol through busking, street begging, borrowing money from friends/family, and selling The Big Issue (a magazine supporting those experiencing homelessness). Such avenues of accessing alcohol were much more difficult during the early pandemic and associated lockdowns:

*When the lockdown first started it was very difficult because nobody was like dropping money and that… [one shopkeeper said] ‘yeah, if you are rough and there is a can’, and it’s not ideal really because that’s just encouraging in a kind of way, but it was helpful. I knew if I was really bad, I could get a can.*

(Client 1)

Clients also described the negative consequences of their drinking, including: seizures from withdrawal; job loss; being the victim of theft when intoxicated; relationship breakdowns; homelessness; involvement with the criminal justice system and spending time in prison; hospitalisations; and being sectioned (compulsorily detained) under the Mental Health Act. All clients discussed issues with their mental health which use of alcohol both exacerbated and acted as a coping mechanism for:

*I use alcohol to block that out… it is a coping mechanism, just coping with daily life. It stops me thinking about my previous life and my thoughts. It blocks it out. Alcohol is my coping mechanism for getting through each day.*

(Client 3)

Participants described the wide range of potential needs for those in a MAP which extended far beyond support for their alcohol use/dependence. It was felt that clients would face a range of complex vulnerabilities in areas such as: physical and mental health; housing and homelessness; trauma; social isolation and disengagement; mistrust of services; and loss of connection with family and children. Such issues would require a holistic and relational approach to supporting clients, based on building non-conditional, trusting relationships over time which challenge the traditional power imbalances of service provider and service recipient:

*What, for me, is a real strong and vital focus on this is not just about the dispensing of alcohol, or the consumption of alcohol, it’s about the relationship that you engage with the person. So, I think irrespective of whether it is a day service or a residential there needs to be a strong and intentional focus on what does a MAP relationship look like?*

(Manager 1)

Participants also felt that, owing to this range of potential needs, a MAP would need to be closely linked to a range of supports and able to tailor support to individual clients. Clients talked about the need to feel supported and not forced to change their drinking or stop drinking, which would be an important part of MAPs:

*You need to speak to people a lot more, like about their alcohol problems, ken [you know], get them in the office, ken [you know] a wee [small] one-to-one, just tell them exactly what you are doing… just say like ‘we are not forcing you or anything we are just trying to help you here’.*

(Client 5)

*You cannot force recovery on anybody, no matter who they are, what they are, where they have been, what they’ve done in their life.*

(Client 6)

Some participants described the current challenges within the sector of being able to access additional support, for example for mental health problems, for those who are seeking help for alcohol dependence, and highlighted the value of MAPs in being able to provide a wide range of holistic, tailored support. It was felt that traditionally for those experiencing alcohol dependence there had been limited support available for those who did not wish, or were unable, to become abstinent. 

#### 3.3.2. Securing Buy-In and Partnership Working

There was a perceived need to secure buy-in from both internal and external stakeholders. Within the organisation (TSA), buy-in was said to be required from senior leadership, staff in services, and clients. Managers noted that those in more senior leadership roles in TSA may need convincing of the need for and value of MAPs because of the traditionally abstinence-based approach of the organisation. Managers involved in the study recognised that, while they as managers were supportive of MAPs, those on the frontline may have different views, so having open and honest conversations about MAPs was important to ensure, “*we bring them alongside us rather than kind of forcing it along*” (Manager 6) prior to implementation. This issue was also reflected in interviews with staff participants who, as noted above, spoke about their concerns, unanswered questions, and particular views on harm reduction. In one instance, a staff participant described their experiences of varying staff views while providing a ‘mini’ MAP with one client over a three-week period, out of necessity to keep them alive, where alcohol was purchased and provided regularly for them:

*What I did notice doing that is, even though both our managers were happy for this mini-MAP to be in place, the rest of the staff team maybe apart from one, were totally against it. They didn’t believe it was right to give someone alcohol. So, I tried to explain it to them: ‘but it’s alright for us to give people needles, through needle exchange, but you are struggling giving people alcohol?’.*

(Staff 7)

Some staff were supportive of MAPs from the beginning, viewing them as being in line with their harm reduction ethos. Others were uncertain and lacked a clear understanding of how a MAP would be delivered in practice or had ethical reservations about the provision of alcohol. There appeared to have been a shift in staff perceptions over the pandemic period, however, as they learned more about MAPs, although many said that they still had lots of unanswered questions:

*I don’t actually have a massive understanding. I was on a wee [small] webinar just at the end of last week. I learned a bit more about it. It was more about the Canadian shape of it and how different MAPs can be for different places… so apart from the fact that there is kind of stats and that and they stats all seem to look good, there is not a lot that we actually know bar that… or how we would implement it and in and about our service.*

(Staff 2)

Participants also discussed the importance of engaging with clients prior to and whilst implementing a MAP, and some staff and managers mentioned that they had already had conversations with clients to try and build their understanding of it. The importance of having support and buy-in from those with lived and living experience was noted by participants, as they were believed to be able to help secure legitimacy and provide powerful stories to support the rationale for the need for MAPs:

*Telling that story and hearing the testimony of individuals who have been through that, is the best way to tell that story and to convince people that this is a positive thing for some people.*

(Stakeholder 1)

Clients themselves also described the importance of involving those with lived experience in the planning and delivery of MAPs, with one client explicitly stating that a MAP should be staffed by, “*ex drinkers*” (Client 6), and another noting, “*it would be good if like with the MAP thing, people have been in it, and got this out of it, this is what you could do*” (Client 1).

Buy-in from staff working in the national health service (NHS) was also viewed as impacting the delivery and governance of MAPs. As previously noted, participants stated that there was a need for rigorous clinical input alongside the logistical complexity of delivering the programme, and the necessary partnership working required between statutory healthcare and third sector (not-for-profit) services. While there was a continuation in the shift towards harm reduction interventions in TSA, a greater openness to alcohol harm reduction interventions was not necessarily universal across the sector, which was viewed as potentially impacting the delivery of MAPs. This was evidenced by one stakeholder who felt that funding would be better directed towards preventive and traditional ‘*proven’* modes of treatment, rather that allocated to MAPs:

*I’m a bit ambivalent about the use of the managed alcohol programme. I would really, if I was advising the National Health Service now, I would say that the funding needs to be increased to prevent people becoming homeless alcoholics… you should fund programmes which have proven effectiveness which intensive alcohol treatment programmes, usually abstinence orientated but not necessarily, but intensive with follow up with Antabuse with group support, with Alcoholics Anonymous.*

(Stakeholder 8)

Participants also discussed the need to build positive relationships with external stakeholders who would be involved in the implementation of MAPs, including those working in health and social care and local and national government. One participant noted that, while TSA already had positive relationships with government bodies, there would be a need to begin having specific conversations about MAPs in order to facilitate implementation. Buy-in from those working in health and social care was also deemed important in terms of MAP delivery, at all levels of the system, from management to frontline staff. There was a view that there would need to be shared understandings between the MAP and local services and that clinicians who are involved need to, *“have confidence in what they are delivering which means you have really good and robust clinical governance systems”* (Stakeholder 18). However, there were concerns that the approach to alcohol within MAPs may conflict with some statutory treatment services which do not allow those under the influence to engage in groups:

*If they were having ten units in a morning, I would probably not let them into a group, if they were presenting as being under the influence because of the risk to other group members and their engagement levels and things like that … people don’t need to be abstinent, but they need to be able to come not under the influence.*

(Stakeholder 19)

Overall, while there was evidence of a changing culture of alcohol harm reduction within the organisation which was not necessarily reflected in the wider field. While MAPs had the potential to address a current gap in service provision, buy-in from a range of stakeholders, internal and external, was thought to be required prior to implementation. While there was general support for MAPs, more buy-in Scotland was deemed to be required if they were to be implemented either during or post pandemic, particularly from frontline staff, senior management, and external stakeholders. Greater awareness of the evidence base and benefits of MAPs within a Scottish context, as well as training and support for staff, were considered to be important for buy-in. Key implementation components of MAPs were identified. Securing funding to enable MAPs to be implemented over the long-term was also deemed essential. There was a view that MAPs would have been able to provide a number of benefits to people during the COVID-19 pandemic, but they were required as a long-term solution and not something that could be hastily implemented. As well as addressing the key considerations, there was a view that a culture shift towards alcohol harm reduction would be required across the sector. 

## 4. Discussion

This paper provides insight into the key areas of implementation required for MAPs in Scotland in the context of the COVID-19 pandemic and beyond. No MAPs were implemented in Scotland during the time the data were collected, although one has subsequently opened. This new MAP is run by another third sector organisation, Simon Community Scotland. We describe key areas for successful implementation of MAPs across three of the five CFIR domains: intervention characteristics, inner setting, and outer setting. Participants reflected on the strong evidence base in Canada compared to the limited evidence from Scotland which was described as impacting potential adoption and wider acceptance. There was also a view that there was a dearth of evidence for MAPs and alcohol harm reduction compared to abstinence-based approaches. 

While there is some evidence for alcohol harm reduction interventions, including MAPs, the evidence base is also lacking for abstinence-based programmes such as Alcoholics Anonymous (AA) and residential treatment in general populations and even less for those experiencing homelessness. One review on residential treatment concluded that it is unclear who residential treatment works best for, why and how [33]. Another review of AA concluded it is effective inn helping people achieve abstinence [34] but issues have been raised regarding the validity of the findings [35]. More work is therefore required in terms of informing stakeholders of the wider evidence base of interventions for alcohol dependence. 

A range of necessary components of MAPs were identified, in terms of service delivery approaches, tailoring to local contexts, staffing, and funding, building on factors identified in previous research [6,26]. Several themes relating to the current climate and environment shed light on the challenges of implementing and delivering alcohol harm reduction in a context traditionally dominated by abstinence-only approaches. MAPs were viewed as a moral and ethical grey area which challenged participants’ views on harm reduction, treatment, and alcohol provision in fundamental ways. Relating to the wider environment, MAPs were viewed as an intervention that would address a service gap for those not wishing to become abstinent. For MAPs to be successful, buy-in and funding will therefore be required from a wide range of stakeholders. 

Our findings provide insight into the key areas of implementation required for developing and delivering MAPs in Scotland, building on the existing evidence base [6,26]. The study was conducted in the early stages of the COVID-19 pandemic in the UK, but many of the key considerations for MAPs highlighted were not directly related to the pandemic. Instead, they were long-term, complex issues that would require months, or years, of planning. While there were examples of MAPs being established during the pandemic, in the US [23,24] and Portugal [36], our study highlights that much work is still required in Scotland, and in our organisation in focus, the TSA, to gain funding, secure buy-in, and determine the most suitable service delivery model(s). Thus, while MAPs would have been a suitable intervention to implement during the early stages of the pandemic, as described in our related paper [9], they are also required as a long-term approach in Scotland to address the lack of service provision for those experiencing alcohol dependence and homelessness. Using the CFIR has enabled us to identify and describe the key components of MAPs as described by our participants, to inform future service delivery.

The findings regarding the moral and ethical challenges of delivering MAPs is an underexplored yet vital dimension of MAPs that needs addressed [37]. Ethical concerns related to harm reduction are deeply embedded in a broader social context and approach to management of substance use and long-standing history of abstinence-only approaches. There is still a tension, within both policy and practice, between abstinence-based and harm reduction approaches [7]. In our study, those working in frontline roles, especially those with lived experience, appeared to struggle with the harm reduction approach being proposed in a traditionally abstinence-based organisation, and questioned the ethics of providing alcohol rather than supporting people to stop drinking. Those working with people experiencing problems with substances are faced with ethical dilemmas and moral tensions on a daily basis as a result of their work [38,39], and it is likely that these are intensified for those whose views may be at odds with the type of service or care being provided. Henwood et al. (2014) note that those providing abstinence-based services should consider whether and how harm reduction can be provided to those they work with, especially individuals who are unable or unwilling to become abstinent [40]. Harm reduction and abstinence-based approaches can be and often are delivered in parallel to meet the wide range of needs and preferences of those experiencing homelessness and alcohol dependence [40,41]. The COVID-19 pandemic facilitated changes towards harm reduction in many areas, with Narasimha et al. (2022) noting the piloting of MAPs in the US as a key example of this [42]. 

Ivsins et al. (2019) highlight the important role of MAPs within the field of harm reduction, which is often dominated by illicit drug harm reduction, and the role of MAPs as part of alcohol policy [43]. Scotland has been a world leader in alcohol policy which aims to reduce harms yet approaches at the organisational level continue to be dominated by abstinence-only approaches. MAPs sit as an important intervention to address unintended harms of both abstinence-only approaches which exclude those who are unable to participate, as well as policy approaches that leave some individuals vulnerable to ongoing harms [43]. Alcohol harm reduction is becoming more widely acknowledged as an important and evidence-based approach for those for whom abstinence is not (yet) an option. Increasing the Scottish evidence base, as our findings highlight, may be particularly beneficial in changing views and securing buy-in, as well as acknowledging the importance of creating shifts in organisational cultures towards harm reduction approaches.

The CFIR provided a useful framework for systematically identifying the key facilitators and barriers to MAPs in Scotland during the COVID-19 pandemic. This approach enabled us to identify the most important factors for consideration in the implementation of MAPs, as well as to identify implications for policy and practice. Our findings highlight the lack of appropriate services for people experiencing alcohol dependence and homelessness in Scotland. MAPs would directly address this service gap over the long-term, but require significant buy-in and resources including long-term funding, to provide well-staffed and well-run services with appropriate clinical input. They require buy-in from those working in the services, those providing support, and those external to the service, including local and national government and the general public. Building the evidence base, and providing a clear rationale for why MAPs are an important and much-needed intervention, should facilitate such buy-in. It is important for frontline staff to be provided with more information regarding MAPs and alcohol harm reduction more generally, in order to alleviate any fears around alcohol provision, as well as clarifying roles/responsibilities. Future research is required to develop the evidence base, including rigorous evaluation of the current MAP in Scotland, and any other services that are established.

### Strengths and Limitations

In terms of strengths, data were collected across four participant groups and within a wide range of settings, which enabled a diverse range of views to be captured. The collaborative partnership between TSA and the research team meant that data could be collected during a very challenging time within services. Being able to collect data from clients was particularly important to ensure that the needs of those who might use MAPs can inform future service delivery. Finally, using the CFIR enabled us to systematically identify the key components required for the implementation of MAPs and identify areas that may have been missed. 

There are also several limitations. Firstly, as noted in our related paper [9], service managers and staff may have been biased in identifying staff and clients to participate in the interviews. While we attempted to capture a range of views via our sampling framework, it is possible that those who participated in the interviews were more aware of, knowledgeable about, and supportive of, MAPs. Secondly, we were limited in the number of interviews conducted, particularly with clients, due to the time limit placed on the study and lockdown restrictions which necessitated remote data collection. Finally, the study was conducted in only one organisation which limits the transferability of the findings. We did, however, include four services in two cities which encompassed different service models, as well as including a range of stakeholders from other relevant organisations across Scotland. 

## 5. Conclusions

The aim of this mixed methods study was to explore the potential utility of MAPs in TSA settings in Scotland during the early COVID-19 pandemic. This paper reported on the qualitative data which was informed by the CFIR. Six themes were identified which mapped onto three of the CFIR domains. There was a view that MAPs are needed in Scotland due to the lack of appropriate alcohol harm reduction services for people experiencing homelessness and alcohol dependence. Several necessary components were identified as important for implementation, including service models, tailoring to the local context, staffing, and funding. Securing buy-in from a range of stakeholders and partnership working, particularly with health and social care organisations, was deemed to be essential. The cultural ethos of abstinence-only approaches was identified as a key challenge to implementation. MAPs were viewed within the context of a changing culture towards alcohol harm reduction and as ethically and morally challenging for some service providers. A careful, long-term approach to planning is therefore required to ensure MAPs meet the needs of clients. MAPs would have been a beneficial intervention in Scotland during the COVID-19 pandemic but are also required as a long-term approach for those experiencing alcohol dependence and homelessness for whom there are limited existing services.

## Figures and Tables

**Table 1:** Participant characteristics.

Participants and Organisations	
*External stakeholders (n = 19)*	
Health	N = 11
Statutory	N = 4
Other	N = 4
*TSA service managers (n = 8)*	
National role	N = 3
Frontline service manager	N = 5
*TSA frontline staff (n = 7)*	
Setting 1	N = 4
Setting 2	N = 2
Setting 3	N = 1
*Potential beneficiaries/clients (n = 6)*	
Setting 1	N = 1
Setting 2	N = 2
Setting 3	N = 3

**Table 2:** Domains, constructs and key findings.

CFIR Domain	CFIR Construct	Key Findings
Intervention characteristics *The main aspects of the intervention within the organisation which are required for successful implementation.*	Evidence strength and quality Relative advantage Adaptability Trialability Complexity Cost	Perceptions of MAPs and the evidence base Necessary components of MAPs
Inner setting The structural, political, and cultural context in which the intervention will be implemented.	Culture Implementation climate Readiness for change	The changing culture of alcohol harm reduction MAPs as a moral and ethical grey area
Outer setting *The economic, social and political context in which the organisation delivering the intervention sits.*	Patient needs and resources External policy and incentives	Addressing a service gap Securing buy-in and partnership working

## Data Availability

The datasets generated and/or analysed during the study are not publicly available. Individual privacy could be compromised if the dataset is shared due to the small sample involved.

## Supplementary Materials

The following supporting information can be downloaded at: https://www.mdpi.com/article/10.3390/ijerph192215207/s1, Supplementary File S1: Interview schedules for all groups.
